# Emergency Department Presentations of Diabetic Ketoacidosis in a Large Cohort of Children

**DOI:** 10.1155/2023/6693226

**Published:** 2023-09-20

**Authors:** Lise E. Nigrovic, Nathan Kuppermann, Simona Ghetti, Jeff E. Schunk, Michael J. Stoner, Arleta Rewers, Julie K. McManemy, Kimberly S. Quayle, Jennifer L. Trainor, Leah Tzimenatos, Jonathan E. Bennett, Maria Y. Kwok, Sage R. Myers, Kathleen M. Brown, T. Charles Casper, Cody S. Olsen, Nicole S. Glaser, for the Pediatric Emergency Care Applied Research Network (PECARN) DKA FLUID Study Group

**Affiliations:** ^1^Division of Emergency Medicine, Department of Pediatrics, Boston Children's Hospital, Harvard Medical School, Boston, MA, USA; ^2^Department of Emergency Medicine, University of California Davis Health, School of Medicine, Sacramento, CA, USA; ^3^Department of Pediatrics, University of California Davis Health, School of Medicine, Sacramento, CA, USA; ^4^Department of Psychology, University of California Davis, Davis, CA, USA; ^5^Department of Pediatrics, University of Utah School of Medicine, Salt Lake City, UT, USA; ^6^Division of Emergency Medicine, Department of Pediatrics, Nationwide Children's Hospital, The Ohio State University School of Medicine, Columbus, OH, USA; ^7^Section of Emergency Medicine, Department of Pediatrics, University of Colorado-Denver, School of Medicine, Aurora, CO, USA; ^8^Division of Emergency Medicine, Department of Pediatrics, Texas Children's Hospital, Baylor College of Medicine, Houston, TX, USA; ^9^Division of Emergency Medicine, Department of Pediatrics, St. Louis Children's Hospital, Washington University School of Medicine in St. Louis, St. Louis, MO, USA; ^10^Division of Emergency Medicine, Department of Pediatrics, Ann & Robert H, Lurie Children's Hospital of Chicago, Northwestern University Feinberg School of Medicine, Chicago, IL, USA; ^11^Division of Emergency Medicine, Nemours Children's Hospital, Sidney Kimmel Medical College at Thomas Jefferson University, Philadelphia, PA, USA; ^12^Division of Emergency Medicine, Department of Pediatrics, New York Presbyterian Morgan Stanley Children's Hospital, Columbia University College of Physicians and Surgeons, New York, NY, USA; ^13^Division of Emergency Medicine, Department of Pediatrics, Children's Hospital of Philadelphia, Perelman School of Medicine at the University of Pennsylvania, Philadelphia, PA, USA; ^14^Division of Emergency Medicine, Department of Pediatrics, Children's National Medical Center, The George Washington School of Medicine and Health Sciences, Washington, DC, USA

## Abstract

**Background:**

Diabetic ketoacidosis (DKA) is a potentially life-threatening complication of childhood diabetes. However, the influence of demographic factors on presentation are not well-defined.

**Methods:**

We included children from 12 centers who were <18 years with DKA (glucose > 300 mg/dL, serum pH < 7.25, or serum bicarbonate <15 mEq/L) enrolled in the Pediatric Emergency Care Applied Research Network (PECARN) Fluid Therapies Under Investigation in DKA (FLUID) Trial. Data were also collected for children who presented to the centers during the enrollment period but were not enrolled due to disease or treatment-related reasons. We compared demographic, clinical, and biochemical findings among children with newly and previously diagnosed diabetes and children in different age groups.

**Results:**

Of the 1,679 DKA episodes in 1,553 children, 799 (47.5%) episodes occurred in children with newly diagnosed diabetes and 396 (23.6%) were severe (pH < 7.1). Newly diagnosed children <6 years of age were not more likely to have severe DKA in terms of pH, but had more severe hypocarbia and higher blood urea nitrogen levels, factors previously associated with the risk of cerebral injury. Lower socioeconomic status (SES) (based on family income and maternal education level) were associated with more severe DKA in new onset children, and recurrent DKA in the previously diagnosed children.

**Conclusions:**

Greater efforts are needed to identify the children with diabetes early and to prevent recurrent DKA, particularly among children in low-SES groups. Young children with DKA may need more intensive monitoring due to higher risk of cerebral injury.

## 1. Introduction

Diabetic ketoacidosis (DKA) is a frequent presentation in children with undiagnosed type 1 diabetes as well as a recognized complication of established diabetes [1]. Even a single DKA episode, especially a severe one, has been associated with a lower intelligence quotient in children with diabetes as well as increased risk of kidney dysfunction, suggesting subtle brain and kidney injury as a result of this metabolic derangement [2, 3]. Therefore, avoidance of any episodes of DKA should be the goal for all children with type 1 diabetes, their care givers, and their medical providers.

Several demographic factors, including age, timing of diabetes diagnosis (new vs. previous), and socioeconomic status (SES) may affect the presentation, treatment, and outcomes of DKA in children [4–6]. As most studies of DKA in children have been single-center, retrospective, and relatively small [1, 7–11], the relationship between patient-level demographic factors and variations in presentation has not been examined in a diverse, prospectively enrolled cohort of children with DKA.

To this end, we describe a planned secondary analysis of a factorial-design clinical trial of fluid infusion protocols for DKA (the Pediatric Emergency Care Applied Research Network (PECARN) FLUID trial) [12], focusing on demographic factors associated with variations in clinical and biochemical presentation, and disease severity. To provide a more complete description of pediatric DKA presentations, we also collected data on children excluded from the parent trial because of clinical or treatment-related factors and added these encounters to the FLUID Trial database. We aimed to characterize the clinical and epidemiological features of DKA in this large multicenter cohort, identify characteristics of presentations associated with age differences and newly diagnosed vs. previously diagnosed diabetes as well as the SES indicators, and identify factors associated with the severe DKA.

## 2. Materials and Methods

### 2.1. Study Population

We performed a planned secondary analysis of data from the PECARN FLUID Trial, a 13-center clinical trial comparing neurological and neurocognitive outcomes among children with DKA treated with fluid infusion protocols that varied in sodium concentration and rate of infusion (protocol details described previously; clinicaltrials.gov number, NCT00629707) [13]. We added encounters for children with DKA who had been excluded from the FLUID Trial for disease-related reasons.

We included children younger than 18 years who presented between February 2011 and September 2016 with an episode of DKA defined as serum glucose >300 mg/dL and either a venous pH < 7.25 or serum bicarbonate <15 mmol/L. For the FLUID Trial, children with underlying conditions that could affect mental status assessment, Glasgow Coma Scale (GCS) scores at presentation ≤11, factors that caused the treating physician to feel a specific fluid protocol was warranted, or those who had received the substantial treatment prior to presentation to the study site were excluded. For the added retrospective cohort, we included DKA episodes for children meeting one or more of the following criteria who had been excluded from the PECARN FLUID Trial (overall *n* = 378): preexisting neurological disease that substantially impacts mental status or neurocognitive exam (*n* = 59), concomitant alcohol or drug use, head trauma, meningitis, or other conditions which might affect neurologic function (*n* = 14), the treating physician believed a specific fluid and electrolyte regimen was warranted (*n* = 261), the patient was given hyperosmolar therapy prior to the arrival (*n* = 8), clinician intent to immediately administer hyperosmolar therapy (*n* = 19), or baseline GCS was ≤11 (*n* = 32). We did not add DKA encounters for the children whose caregivers refused to participate in the FLUID study or who presented when study staff were not available for the enrollment. Children with more than one eligible episode of DKA during the study period could be included at most twice.

As one of the 13 PECARN FLUID Trial sites was unable to collect the additional retrospective data needed to complete the cohort for the current analysis, all data from that site were excluded from this analysis (*n* = 87 DKA episodes).

### 2.2. Data Collection in the Prospective FLUID Trial

Treating clinicians completed study case report forms that included clinical symptoms and diabetes history. Laboratory results were abstracted from the medical record. Caregivers of children enrolled in the FLUID trial completed surveys during the index hospitalization that included the caregiver's highest education achieved (categorized for analysis as high school/GED or less, some college/vocational school, college degree, or more) as well as annual household income (categorized for analysis as less than $40,000, $40,000–$99,999 and $100,000 or more).

### 2.3. Data Collection for the Retrospective Cohort

For the additional 378 DKA episodes included in the retrospective cohort but not in the FLUID trial, study teams completed structured medical record reviews using REDCap™ hosted by the PECARN data center at the University of Utah [14]. Patient demographics and clinical factors were abstracted from the medical records. Caregiver education and annual household income were not available for this cohort.

### 2.4. DKA Severity

We defined DKA severity using the first venous pH obtained, applying standard definitions: mild (pH ≥ 7.2–<7.25), moderate (pH ≥ 7.1–<7.2), and severe (pH < 7.1) [15]. For children who did not have pH measured before starting treatment, we classified DKA severity according to the serum bicarbonate concentration as mild (bicarbonate ≥ 10), moderate (bicarbonate 5–<10), and severe (bicarbonate < 5).

### 2.5. Initial Laboratory Results

Initial laboratory results were defined as the earliest result measured at the participating emergency department for both children enrolled in the FLUID trial or included in the retrospective cohort. Sodium values were corrected for the glucose according to the following formula: corrected sodium concentration = measured sodium concentration +1.6 ((blood glucose−100)/100) [16]. Creatinine values were adjusted for the age using *z*-scores based on age-specific median creatinine estimates [17]. These scores represent how many standard units each measurement was above or below the reference creatinine value.

### 2.6. Cerebral Injury Frequency

Every DKA episode in which a child received therapy for cerebral injury (i.e., mannitol, hypertonic saline, and/or endotracheal intubation) or in which the child died was adjudicated by expert review to determine whether clinical criteria for diagnosis of DKA-related cerebral injury were met. For participants enrolled in the FLUID trial, the review was done by a panel of experts. For the supplemental data added to the current study, the study endocrinologist (NSG) adjudicated the cases, using the same criteria as those used by the expert panel. A child was considered to have had a clinically apparent cerebral injury when symptoms and findings met criteria defined in established diagnostic algorithms [18, 19].

### 2.7. Statistical Analysis

We included both prospectively and retrospectively collected data in the analytic database.

The unit of analysis was the DKA episode, with up to two eligible DKA encounters per patient during the study period. We compared presenting demographic, clinical, and biochemical findings among children by diabetes history (newly vs. previously diagnosed). Then we compared DKA encounters by age group (0–<6 years, 6–<12 years, and 12–<18 years) and DKA severity (mild, moderate, and severe) stratified by diabetes history. We summarized categorical characteristics using relative frequencies and continuous values using medians and interquartile ranges. We compared characteristics between groups using Fisher's exact and Kruskal–Wallis tests for the categorical and continuous characteristics, respectively. Maternal education and household income were investigated among FLUID trial DKA episodes only. No adjustments were made for multiple testing and a significance level of 0.05 was used for all the tests. Statistical analyses were performed using SAS Software (version 9.4; SAS Institute, Cary, NC).

## 3. Results

**Figure 1:**
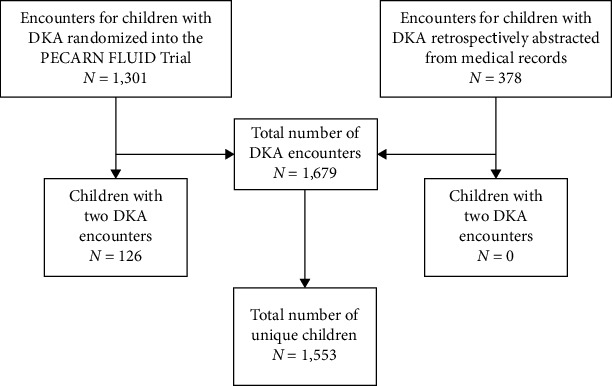
Study participant flow diagram.

Over the 5-year study period, we identified 1,679 DKA episodes for 1,553 unique children treated in one of 12 participating PECARN emergency departments (Figure 1). Of these episodes, 1,301 (77.5% of all included DKA episodes) were included in the FLUID trial. Most children included in the database had a single DKA episode (*n* = 1,427; 91.9%), and 126 (8.1%) had two DKA episodes. The median patient age was 12 years (interquartile range (IQR) 9–14 years) and 781 (46.5%) were boys.

Overall, 799 (47.6%) DKA episodes occurred in children with newly diagnosed diabetes and 395 (23.5%) had severe DKA (pH < 7.1). Fifty children were younger than 2 years (6.3% of those with newly diagnosed diabetes), the median pH in this group was 7.19 (IQR 7.11–7.25), and the median initial serum glucose concentration was 566 mg/dL (IQR 483–627).

Of the 1,679 DKA episodes, 55 (3.3%) received the following treatments for cerebral injury: mannitol 38 (2.3%), hypertonic saline 24 (1.4%), and/or endotracheal intubation 9 (0.5%). After expert case review, 34 (2.0%) of the included DKA episodes were found to meet published criteria for clinically apparent cerebral injury [18]. Eight-one children (5.4% of those with documented GCS scores) had GCS scores < 14 at presentation. Of the 348 DKA episodes for children who reported headaches, 11 (3.2%) received any treatment for cerebral injury. The rate was similar among the 1,331 DKA episodes for children who did not report headaches, in whom 44 (3.3%) received treatment for cerebral injury (*p*=1.0). Six (1.7%) DKA episodes with a headache, and 28 (2.1%) without a headache met criteria for clinically-apparent cerebral injury (*p*=0.83). All but two children (99.9% of study participants) survived to hospital discharge. The two children died from cerebral injury: a 5-year-old with newly diagnosed diabetes (from the FLUID study) and a 12-year-old with previously diagnosed diabetes (from the retrospective cohort).

Children with DKA and newly diagnosed diabetes were younger than those with previously diagnosed diabetes. Although hypokalemia at presentation was uncommon and may require treatment modifications, more children with new onset vs. previously diagnosed diabetes presented with hypokalemia (1.4% newly vs. 0.1% previously diagnosed; *p*=0.002). Children with previously diagnosed diabetes and DKA were more likely to have lower maternal education and household income (Table 1). More than half of children with previously diagnosed diabetes and DKA had household incomes of less than $40,000 per year.

We compared patient age groups stratified by newly diagnosed and previously diagnosed diabetes (Table 2). Children younger than 6 years with both newly and previously diagnosed diabetes were less likely to report headaches at presentation (3% of those <6 years, 16% of those 6–<12 years, and 28% of those 12–<18 years). Children younger than 6 years were not more acidotic than older children, but had lower pCO_2_ levels. Laboratory profiles in the younger children with newly diagnosed diabetes also suggested greater renal dysfunction in this group (higher glucose, potassium and BUN levels with similar creatinine levels despite smaller body size) [20].

We also investigated factors associated with mild, moderate, and severe DKA stratified by newly vs. previously diagnosed diabetes (Table 3). Although age was not associated with severe DKA for those with newly diagnosed diabetes, older age was associated with severe DKA for children with previously diagnosed diabetes. Lower maternal education and lower household income were associated with severe DKA for children with newly diagnosed, but not established diabetes.

## 4. Discussion

In this study, we describe demographic, clinical, and biochemical findings in a large cohort of children with DKA from 12 clinical centers in the United States participating in PECARN FLUID Trial. When we compared age groups, we found that younger children did not have more severe DKA but tended to have greater risk factors for cerebral injury (higher BUN and lower pCO_2_) and greater renal dysfunction despite similar levels of acidosis compared to the other age groups. One of the two children who died from cerebral injury was <6 years of age. Our findings support the recent DKA consensus practice guideline that recommends more vigilant monitoring of very young children with DKA due to the increased risk for cerebral injury [21, 22]. Increased risk of cerebral injury in this age group has been thought to result from greater severity of DKA, although our data suggest that this is not the case. Instead, very young children tend to have more cerebral injury risk factors despite similar levels of acidosis [23].

We also found that children with lower SES were overrepresented among the group with previously diagnosed diabetes. Associations between lower SES and increased frequency of developing DKA have been previously reported [24]. A national cross-sectional study of children with diabetes found that geographic measures of social deprivation based on the residential zip codes were associated with developing DKA [25]. Our emergency department-based DKA cohort demonstrated similar associations. However, our cohort is unique in that the majority of the included DKA episodes were enrolled prospectively allowing standardized data collection of patient and family characteristics. We found that lower maternal educational level and lower household income were associated with severe DKA.

To our knowledge, the frequency of headache at presentation of DKA has not previously been investigated. We found that headache was a common symptom at presentation for children with DKA. Not surprisingly, the frequency of headache increased with age. Younger children have more limited capacity to describe symptoms such as headache, potentially explaining the reduced frequency. Although headache has been reported as a warning sign for the cerebral injury [26], children reporting headache rarely developed clinically apparent cerebral injury during DKA treatment in this study and children with and without headache at presentation had nearly identical rates of development of cerebral injury. Our findings should inform the level of clinical concern for the cerebral injury when children with DKA present with this symptom.

The high frequency of DKA in children with type 1 diabetes in the United States [27, 28] is of concern, particularly in light of accumulating evidence that DKA episodes may increase risk of long-term complications including increased risk of diabetic kidney disease and alterations in cognition. Studies demonstrate that nations with a higher background prevalence of type 1 diabetes tend to have lower rates of DKA at diagnosis, suggesting that increased awareness of DKA may reduce its incidence [2, 3]. Campaigns to increase diabetes awareness may reduce the frequency of DKA at the time of diabetes diagnosis [29]. One particularly successful effort in Italy was able to reduce rates of DKA in people with diabetes from 78% to 13% by placing educational materials in primary care clinics and schools [30]. Targeted monitoring of children at heightened risk for type 1 diabetes (children with first degree relatives with type 1 diabetes, positive pancreatic autoantibodies, or high-risk HLA profiles) have also been highly successful in preventing DKA at the time of diabetes diagnosis in these groups [31–34].

Our findings need to be interpreted in the context of some limitations. First, we were unable to include all episodes of DKA over the study period including those whose caregivers refused study participation and we know that parents of children with previously diagnosed diabetes were more likely to consent to study participation [35]. However, we included both DKA episodes included in the parent FLUID trial as well as additional DKA episodes during the same time-period at the same centers in which the patient was excluded for disease-related reasons. Second, one study site was unable to participate in the medical record review, so we excluded all DKA episodes from that study site for this analysis. However, the children enrolled at that site were similar to the others in the FLUID trial (data not shown). Third, we were unable to determine the timing of the laboratory studies relative to treatment initiation in the retrospective cohort. However, given that the cohorts were treated at the same PECARN hospitals over the same time-period, we believe that the timing of the laboratory tests was similar for the two study cohorts. Additionally, we reported laboratory results at presentation and did not present changes with DKA treatment (e.g., expected drops in potassium with correction of metabolic acidosis) [36, 37]. Fourth, we were unable to abstract SES indicators (either household income or highest caregiver education) for the retrospective cohort from existing medical records, limiting our ability to evaluate these demographic factors in the full study cohort. We chose not to include race or ethnicity as covariates as income and education are better correlates of health outcomes [38]. Future prospective studies of children with DKA need to enroll the most comprehensive and diverse population possible.

## 5. Conclusions

Greater efforts are needed for early identification of diabetes and prevention of DKA, as well as the prevention of recurrent DKA in children with known diabetes. Children from lower SES groups are at greater risk for DKA after initial diagnosis of diabetes and at greater risk for more severe DKA, such that prevention efforts should be particularly targeted toward these children. Biochemical profiles of very young children with DKA also suggest that they are at greater risk of cerebral injury and renal dysfunction, despite similar DKA severity (based on pH) compared to the older age groups. Current guidelines suggesting that more intensive monitoring of very young children during DKA treatment are supported by these findings.

## Figures and Tables

**Table 1:** Comparison between DKA episodes in children with newly vs. previously diagnosed diabetes.

	Diabetes history		*p*-Value
	Newly diagnosed (*N* = 799)	Previously diagnosed (*N* = 880)	
Age (years)	10.0 (6.0, 12.0)	13.0 (11.0, 15.0)	<0.001
Male sex^1^	388 (48.6)	393 (44.7)	0.12
Maternal parental education^1,2^			<0.001
High school/GED or less	157 (26.9)	241 (39.1)	
Some college/vocational school	159 (27.3)	224 (36.3)	
College degree or more	267 (45.8)	152 (24.6)	
Household income^1,2^			<0.001
Less than $40,000	182 (31.4)	342 (53.2)	
$40,000–$99,999	209 (36.1)	218 (33.9)	
$100,000 or more	188 (32.5)	83 (12.9)	
Clinical characteristics			
Headache at presentation^1^	95 (11.9)	253 (28.8)	<0.001
Clinically apparent cerebral injury^1^	15 (1.9)	19 (2.2)	0.73
Laboratory results (baseline)^3^			
BUN (mg/dL)	13 (10, 17)	19 (15, 22)	<0.001
Bicarbonate (mEq/L)	9 (6, 11)	9 (6, 12)	0.01
Creatinine *Z*-Score for age (standard units)	0.3 (−0.1, 0.8)	0.6 (0.3, 1.2)	<0.001
Glucose (mg/dL)	519 (425, 615)	495 (400, 600)	<0.001
Potassium (mEq/L)	4.4 (3.9, 5.0)	5.1 (4.6, 5.7)	<0.001
Sodium corrected (mEq/L)	140 (137, 144)	142 (138, 145)	<0.001
pCO_2_ (mm Hg)	24 (20, 29)	27 (22, 32)	<0.001
pH	7.20 (7.11, 7.26)	7.17 (7.10, 7.23)	<0.001

^1^ *N* (%) and *p*-values from Fisher's exact tests are presented for categorical characteristics; median (25th and 75th percentiles) and *p*-values from Wilcoxon rank sum tests are presented for all other characteristics. ^2^Maternal parental education and household income information were not collected for the retrospective cohort; number nonmissing and analyzed were 583 newly diagnosed and 617 previously diagnosed for maternal parental education, and 579 newly diagnosed and 643 previously diagnosed for household income. ^3^Baseline laboratory values were missing as follows (newly diagnosed/previously diagnosed): BUN (49/48), bicarbonate (40/28), creatinine (48/52), glucose (19/7), potassium (35/22), sodium (36/23), pCO_2_ (55/40), and pH (51/37).

**Table 2:** Comparison by patient age group stratified by diabetes history.

Newly diagnosed diabetes	Age group (in years)			*p*-Value
	0 to less than 6	6 to less than 12	12 to less than 18	
Number of episodes	184	360	255	
Male sex^1^	87 (47.3)	155 (43.1)	146 (57.3)	0.002
Maternal parental education^1,2^				0.39
High school/GED or less	34 (27.2)	76 (27.0)	47 (26.7)	
Some college/vocational school	38 (30.4)	82 (29.1)	39 (22.2)	
College degree or more	53 (42.4)	124 (44.0)	90 (51.1)	
Household income^1,2^				0.47
Less than $40,000	41 (33.6)	91 (32.9)	50 (27.8)	
$40,000 to $99,999	45 (36.9)	102 (36.8)	62 (34.4)	
$100,000 or more	36 (29.5)	84 (30.3)	68 (37.8)	
Clinical characteristics				
Headache at presentation^1^	3 (1.6)	52 (14.4)	40 (15.7)	<0.001
Clinically apparent cerebral injury^1^	5 (2.7)	10 (2.8)	0 (0.0)	0.009
Laboratory results (baseline)^3^				
BUN (mg/dL)	15 (11, 20)	13 (10, 17)	12 (9, 16)	<0.001
Bicarbonate (mEq/L)	8 (5, 11)	8 (6, 11)	9 (7, 12)	0.02
Creatinine Z-score (standard units)	0.2 (0.0, 0.6)	0.2 (−0.1, 0.7)	0.3 (0.0, 0.9)	0.07
Glucose (mg/dL)	554 (468, 626)	529 (435, 627)	474 (380, 600)	<0.001
Potassium (mEq/L)	4.7 (4.2, 5.1)	4.4 (3.9, 4.9)	4.2 (3.7, 4.8)	<0.001
Sodium corrected (mEq/L)	139 (136, 145)	140 (137, 144)	140 (138, 145)	0.16
pCO_2_ (mm Hg)	23 (18, 27)	24 (20, 28)	26 (22, 30)	<0.001
pH	7.20 (7.09, 7.27)	7.20 (7.11, 7.26)	7.20 (7.12, 7.26)	0.79

Previously diagnosed diabetes				

Number of episodes	29	223	628	
Male sex^1^	20 (69.0)	110 (49.3)	263 (41.9)	0.004
Maternal parental education^1,2^				0.82
High school/GED or less	6 (31.6)	60 (37.3)	175 (40.0)	
Some college/vocational school	9 (47.4)	58 (36.0)	157 (35.9)	
College degree or more	4 (21.1)	43 (26.7)	105 (24.0)	
Household income^1,2^				0.47
Less than $40,000	11 (57.9)	87 (52.4)	244 (53.3)	
$40,000 to $99,999	8 (42.1)	59 (35.5)	151 (33.0)	
$100,000 or more	0 (0.0)	20 (12.0)	63 (13.8)	
Clinical characteristics				
Headache at presentation^1^	4 (13.8)	43 (19.3)	206 (32.8)	<0.001
Clinically apparent cerebral injury^1^	2 (6.9)	4 (1.8)	13 (2.1)	0.19
Laboratory results (baseline)^3^				
BUN (mg/dL)	20 (15, 23)	20 (17, 25)	18 (14, 22)	<0.001
Bicarbonate (mEq/L)	10 (7, 12)	10 (7, 13)	8 (6, 12)	0.003
Creatinine Z-score (standard units)	0.4 (0.3, 0.6)	0.7 (0.3, 1.2)	0.6 (0.3, 1.2)	0.30
Glucose (mg/dL)	376 (350, 527)	485 (392, 600)	500 (406, 600)	0.004
Potassium (mEq/L)	4.9 (4.4, 5.5)	5.1 (4.7, 5.7)	5.1 (4.6, 5.7)	0.26
Sodium corrected (mEq/L)	140 (137, 143)	141 (138, 144)	142 (139, 145)	0.007
pCO_2_ (mm Hg)	25 (22, 31)	29 (24, 34)	27 (22, 32)	0.002
pH	7.22 (7.13, 7.28)	7.20 (7.13, 7.23)	7.16 (7.08, 7.23)	<0.001

^1^ *N* (%) and *p*-values from Fisher's exact tests are presented for categorical characteristics; median (25th and 75th percentile) and *p*-values from Wilcoxon rank sum tests are presented for all other characteristics. ^2^Maternal parental education and household income information were not collected for the retrospective cohort; number nonmissing and analyzed were (0–<6/6–<12/12–<18 years): (125/282/176) newly diagnosed and (19/161/437) previously diagnosed for maternal parental education, and (122/277/180) newly diagnosed and (19/166/458) previously diagnosed for household income. ^3^Baseline laboratory values were missing as follows (newly diagnosed 0–<6/6–<12/12–<18 years; previously diagnosed 0–<6/6–<12/12–<18 years): BUN (15/24/10; 2/17/29), bicarbonate (16/16/8; 1/8/19), creatinine (14/24/10; 3/18/31), glucose (5/8/6; 0/2/5), potassium (11/18/6; 0/8/14), sodium (10/16/10; 0/7/16), pCO_2_ (13/25/17; 1/10/29), pH (13/22/16; 0/11/26).

**Table 3:** Comparison by DKA severity stratified by diabetes history.

Newly diagnosed diabetes	DKA severity^1^			*p*-Value
	Mild	Moderate	Severe	
Number of episodes	408	209	181	
Age (years)^2^	10.0 (6.0, 12.0)	10.0 (7.0, 12.0)	9.0 (5.0, 12.0)	0.58
Male sex	202 (49.5)	108 (51.7)	78 (43.1)	0.21
Maternal parental education^3^				0.03
High school/GED or less	77 (25.8)	38 (23.3)	42 (34.7)	
Some college/vocational school	77 (25.8)	43 (26.4)	39 (32.2)	
College degree or more	145 (48.5)	82 (50.3)	40 (33.1)	
Household income^3^				0.02
Less than $40,000	79 (26.5)	59 (36.2)	44 (37.3)	
$40,000 to $99,999	104 (34.9)	60 (36.8)	45 (38.1)	
$100,000 or more	115 (38.6)	44 (27.0)	29 (24.6)	
Clinical characteristics				
Headache at presentation	37 (9.1)	35 (16.7)	23 (12.7)	0.02
Clinically apparent cerebral injury	0 (0.0)	2 (1.0)	13 (7.2)	<0.001

Previously diagnosed diabetes				

Number of episodes	355	311	214	
Age (years)^2^	13.0 (10.0, 15.0)	13.0 (11.0, 15.0)	14.0 (12.0, 16.0)	<0.001
Male sex	156 (43.9)	144 (46.3)	93 (43.5)	0.77
Maternal parental education^3^				0.13
High school/GED or less	82 (35.0)	91 (37.6)	68 (48.2)	
Some college/vocational school	94 (40.2)	87 (36.0)	43 (30.5)	
College degree or more	58 (24.8)	64 (26.4)	30 (21.3)	
Household income^3^				0.07
Less than $40,000	113 (47.5)	134 (53.0)	95 (62.5)	
$40,000 to $99,999	90 (37.8)	85 (33.6)	43 (28.3)	
$100,000 or more	35 (14.7)	34 (13.4)	14 (9.2)	
Clinical characteristics				
Headache at presentation	94 (26.5)	88 (28.3)	71 (33.2)	0.22
Clinically apparent cerebral injury	1 (0.3)	3 (1.0)	15 (7.0)	<0.001

^1^DKA severity was defined using the first venous pH obtained: mild (pH > 7.2 to <7.25), moderate (pH > 7.1 to <7.2), and severe (pH < 7.1). For children with no pH documented prior to treatment, serum bicarbonate concentration was used: mild (bicarbonate ≥ 10), moderate (bicarbonate 5 to <10), and severe (bicarbonate < 5). DKA severity was unknown for one newly diagnosed child. ^2^Mean (25th and 75% percentile) and *p*-values from Wilcoxon rank sum tests are presented for age; *n* (%) and *p*-values from Fisher's exact tests are presented for all other characteristics. ^3^Maternal parental education and household income information were not collected for the retrospective cohort; number nonmissing and analyzed were (mild/moderate/severe DKA): (299/163/121) newly diagnosed and (234/242/141) previously diagnosed for maternal parental education, and (298/163/118) newly diagnosed and (238/253/152) previously diagnosed for household income.

## Data Availability

The study protocol, manual of operations, annotated case report forms, dataset summaries, and instructions for obtaining access to deidentified individual participant data for the prospective FLUID trial are publicly available at https://pecarn.org/datasets.
